# mTOR pathway somatic variants and the molecular pathogenesis of hemimegalencephaly

**DOI:** 10.1002/epi4.12377

**Published:** 2020-01-26

**Authors:** Camila A. B. Garcia, Simone C. S. Carvalho, Xiaoxu Yang, Laurel L. Ball, Renee D. George, Kiely N. James, Valentina Stanley, Martin W. Breuss, Ursula Thomé, Marcelo V. Santos, Fabiano P. Saggioro, Luciano Neder Serafini, Wilson A. Silva, Joseph G. Gleeson, Hélio R. Machado

**Affiliations:** ^1^ Department of Surgery and Anatomy Ribeirão Preto Medical School University of São Paulo (USP) Ribeirao Preto SP Brazil; ^2^ Department of Genetics Ribeirão Preto Medical School University of São Paulo (USP) Ribeirao Preto SP Brazil; ^3^ Laboratory for Pediatric Brain Disease Howard Hughes Medical Institute Department of Neurosciences University of California San Diego, La Jolla CA USA; ^4^ Department of Neurosciences and Behavioral Sciences Ribeirão Preto Medical School University of São Paulo (USP) Ribeirao Preto SP Brazil; ^5^ Department of Pathology Ribeirão Preto School of Medicine University of São Paulo USP Ribeirao Preto SP Brazil; ^6^ Center for Medical Genomics University Hospital of Ribeirão Preto Medical School (USP) Ribeirao Preto SP Brazil

**Keywords:** epilepsy, hemimegalencephaly, mTOR

## Abstract

**Objectives:**

Recently, defects in the protein kinase mTOR (mammalian target of rapamycin) and its associated pathway have been correlated with hemimegalencephaly (HME). mTOR acts as a central regulator of important physiological cellular functions such as growth and proliferation, metabolism, autophagy, death, and survival. This study was aimed at identifying specific variants in mTOR signaling pathway genes in patients diagnosed with HME.

**Methods:**

Using amplicon and whole exome sequencing (WES) of resected brain and paired blood samples from five HME patients, we were able to identify pathogenic mosaic variants in the mTOR pathway genes *MTOR*, *PIK3CA*, and *DEPDC5*.

**Results:**

These results strengthen the hypothesis that somatic variants in PI3K‐Akt‐mTOR pathway genes contribute to HME. We also describe one patient presenting with a pathogenic variant on *DEPDC5* gene, which reinforces the role of *DEPDC5* on cortical structural changes due to mTORC1 hyperactivation. These findings also provide insights into when in brain development these variants occurred. An early developmental variant is expected to affect a larger number of cells and to result in a larger malformation, whereas the same variant occurring later in development would cause a minor malformation.

**Significance:**

In the future, numerous somatic variants in known or new genes will undoubtedly be revealed in resected brain samples, making it possible to draw correlations between genotypes and phenotypes and allow for a genetic clinical diagnosis that may help to predict a given patient's outcome.

**Key Points**

- HME patients typically present with refractory epilepsy, which may or may not be associated with focal neurological deficits and delayed neuropsychomotor development of varying severity.
- Histological and immunohistochemical analyses usually show disorganized cortical lamination, presence of balloon cells, cytomegalic neurons, dysmorphic neurons, ectopic neurons, polymicrogyria and intensely marked astrocytes with hypertrophic processes.
- The finding that *DEPDC5 *is associated with cases of HME following hereditary focal epilepsy clarifies the evidence that *DEPDC5 *acts as an inhibitor of mTORC1, and variants in *DEPDC5 *may be responsible for structural changes in the cerebral cortex with the presence of dysmorphic cells due hyperactivation of mTORC1.
- The characterization of variants in mTOR pathway genes raises the possibility that mTOR inhibitors could have wider therapeutic benefit for patients with focal epilepsy.

## 1 INTRODUCTION

Hemimegalencephaly (HME) is a rare malformation of cerebral cortical development characterized by enlargement of an entire cerebral hemisphere due to defects in neuronal migration and proliferation defects.^1^, ^2^ The histopathological features of HME resemble focal cortical dysplasia (FCD) type II but, unlike FCD, the lesion extends to the whole hemisphere, resulting in unilateral enlargement. Anatomically, areas of polymicrogyria, pachygyria, and heterotopia may also be present.^3^

Clinically, HME patients typically present with refractory epilepsy, which may or may not be associated with focal neurological deficits and delayed neuropsychomotor development of varying severity. They are frequently referred for surgical treatment, with either hemispherectomy or, more often, resection being the procedure of choice.^2^, ^3^ However, satisfactory control of seizures may not be achieved in some HME patients, even after all treatments have been instituted. Thus, genetic and molecular analysis is an important tool to help further understand the molecular and cellular pathogenesis of this disease and the potential viability of therapeutic alternatives. A genetic etiology for HME has long been hypothesized, and recently, somatic variants in genes of the PI3K‐Akt‐mTOR cell signaling pathway, such as *AKT3, PIK3CA, MTOR*, and *DEPDC5*, have been reported.^4^, ^5^, ^6^, ^7^, ^8^

The aim of this study was to identify genetic variants in mTOR signaling pathway genes as well as other parallel pathways. We analyzed DNA from resected brain tissue and paired blood samples of five HME patients who underwent surgical treatment. We identified pathogenic and likely pathogenic variants in several mTOR pathway genes including *DEPDC5, PIK3CA*, and *MTOR*. Our results confirm different manifestations of aberrant mTOR signaling, with complex combinations of mosaic variants, suggesting that therapies targeting this pathway may prove useful across a range of cortical development malformations.

## 2 SUBJECTS/MATERIALS AND METHODS

### 2.1 Patient cohort

Five subjects clinically diagnosed with HME were enrolled in this study. They were all under the age of 7 years at the time of referral and underwent cortical resection procedures to treat refractory epilepsy at the University Hospital of Ribeirão Preto Medical School (HCFMRP‐USP). The surgical strategy was decided after an extensive presurgical workup, including magnetic resonance imaging (MRI) scans and video‐electroencephalography (EEG) in order to accurately localize the epileptogenic zone. After evaluating all clinical and electroencephalographic data, if a focal, regional, or hemispheric epileptogenic zone was identified, the surgical procedure was proposed.

For each subject, we collected a thorough clinical history and obtained pre‐ and postoperative brain scans, as well as formalin‐fixed and paraffin‐embedded affected brain tissue sections stained with neural nuclei (NeuN), glial fibrillary acidic protein (GFAP) and hematoxylin and eosin (H&E), histopathological reports, and Engel surgical outcome scores at one‐year follow‐up.

Brain samples were obtained via biopsy of brain tissue, as per determined by preoperative scans, and further checked during the surgical procedure with the aid of a neuronavigation stealth system. The biopsied tissue was then fractionated into two parts, one of which was sent for anatomopathological study and another for molecular analysis. Venous blood samples were also collected at the time of surgery.

The protocol for this study received prior approval from the Research Ethics Committee of HCFMRP‐USP (Reference Number 6978/2015), and informed consent was obtained from all patients.

### 2.2 Pathology and Immunohistochemistry

The anterior brain portions were cut into 5‐µm‐thick coronal sections and mounted on histological slides, which were stained with hematoxylin‐eosin (H&E), in order to evaluate general cellular architectural patterns, distribution of structures, and cellular density.

Glial fibrillary acidic protein (GFAP) assays were performed to assess the distribution and morphological aspects of astrocytes, and NeuN was used to analyze the arrangement of neurons in columns and layers. Spring Bioscience polymer was applied, and slices were incubated overnight at 4°C with the respective primary anti‐GFAP antibody 1:2000 (anti‐GFAP (DAKO 6F2™)) and anti‐NeuN antibody 1:750 (anti‐NeuN (CHEMICON A60™)). They were visualized with 3,3′‐diaminobenzidine (DAB; Sigma). Next, slides were counter‐colored with hematoxylin, washed under running water, dehydrated by serial crescent ethanol solutions and xylol, and covered with Permount™ cover glasses. Photographic documentation was made with an Axioskop2 plus light microscope (Carl Zeiss™) and an AxioCamHrc digital camera (Carl Zeiss), connected to a computer equipped with AxioVision 3.1 software (Carl Zeiss). A 40× objective was used for GFAP and NeuN immunohistochemistry analysis, and a 10× objective was used for H&E stain.^1^

### 2.3 Whole exome sequencing, screening for variants, and validation of mTOR pathway variants

Brain tissue and whole blood‐derived DNA samples were subjected to exome capture. For this, DNA was extracted using the DNA Purification Kit (Promega) according to the manufacturer's instructions. Libraries were prepared from 250 ng genomic DNA, and exonic regions were captured by using the Nextera Rapid Capture Exome Kit (Illumina). Targeted amplicon sequencing libraries were also carried out for the genomic region of *MTOR, PIK3CA, TSC1, TSC2, DEPDC5,* and *AKT*.

Libraries of 125‐bp paired‐end reads were sequenced on a HiSeq 2500 sequencer (Illumina) with V1 or V4 kits, to achieve a mean coverage of 250× for brain samples and 121× for blood samples. WES and amplicon data were obtained for three patients, whereas two patients had amplicon data only. The variants were filtered, and the variants found by WES were validated using droplet digital PCR (ddPCR).

A mix of ddPCR Supermix (Bio‐Rad), mutant and reference probes (0.25 µmol/L each), forward and reverse primers (0.9 µmol/L each), and 30 ng of sample DNA was emulsified into 20 000 droplets using a QX200 Droplet Generator (Bio‐Rad). PCR amplification was performed using the following cycles: 10 minutes at 95°C, 40 cycles of 30 seconds at 94°C and 60 seconds at 60°C, 10 minutes at 98°C. Samples were analyzed using QX200 Droplet Reader and QuantaSoft software (Bio‐Rad). For all samples, we also screened hotspot variants in *PIK3CA* and *DEPDC5* using ddPCR.

### 2.4 Bioinformatics analysis

#### 2.4.1 WES read mapping and filtering

Whole exome sequencing reads from paired brain and blood samples were aligned to the hg19 version of the human reference genome with decoy sequences using bwa‐mem with default parameters. Duplicates were marked with Picard's MarkDuplicates (v1.128, ://broadinstitute.github.io/picard), indels were realigned using GATK’s IndelRealigner (v3.5),^9^ and base quality was recalibrated with GATK, following the GATK 3.5 best practice.

#### 2.4.2 Amplicon read mapping and filtering

Amplicon sequencing for paired brain and blood samples was performed with random hexamers as unique molecular identifiers (UMIs) to tag individual DNA fragments, and PCR duplicates were removed. UMIs were first removed from amplicon sequencing reads and appended to the read name using umi_tools extract.^10^ These reads were then aligned to the hg19 version of the human reference genome with decoy sequences using bwa‐mem with default parameters.^9^ To remove PCR duplicates, reads were grouped based on their mapped location and UMI, and filtered to yield only one read pair per group using umi_tools dedup. Finally, indels were realigned using GATK’s IndelRealigner (v3.5).^9^

#### 2.4.3 Variant calling and identification of brain‐specific somatic variants

To identify known brain‐specific somatic variants, we first compiled a list of variants in mTOR pathway genes, which have been previously associated with cancer or HME cases.^4^, ^5^, ^7^ We then tabulated the number of read pairs that contained the reference and alternate base at each of these alleles. If a brain sample had at least two alternate reads at one of these known alleles, we treated it as a candidate brain‐specific somatic variant.^11^

We called novel brain‐specific somatic variants using two somatic variant callers: Strelka2 (v2.7.0)^11^ and muTect2 (v2).^10^ For each individual, we considered the brain sample as the “tumor” and the blood sample as the “normal.” We generated high‐quality brain‐specific somatic calls by taking the intersection of variants identified by both Strelka2 and muTect2. We annotated these calls with protein consequence (eg, synonymous and noncoding) and allele frequencies from the Exome Aggregation Consortium (ExAC) using the SnpEff software (http:/http://snpeff.sourceforge.net).^12^

We further filtered these variants to include only those that resulted in an amino acid change or protein truncation (ie, missense, stop gain, splice site) and that had an ExAC allele frequency <1%. Finally, we annotated the remaining variants in a tab‐separated table generated using the SnpEff software and scripts. The variants were annotated with functional consequences using the Ensembl Variant Effect Predictor (VEP). Functional consequences of the variants were evaluated based on the following criteria: (a) Variants occurred in protein‐coding regions and canonical splice sites of known FCD‐associated; (b) variants were absent from the ExAC database; (c) variants disrupted highly conserved amino acid residues and were predicted to be deleterious by SIFT (score < 0.05) and damaging or probably damaging by PolyPhen (score > 0.85); and (d) variants were predicted to be disease‐causing by MutationTaster.

## 3 RESULTS

### 3.1 Clinical features and genetic analysis

**Figure 1:**
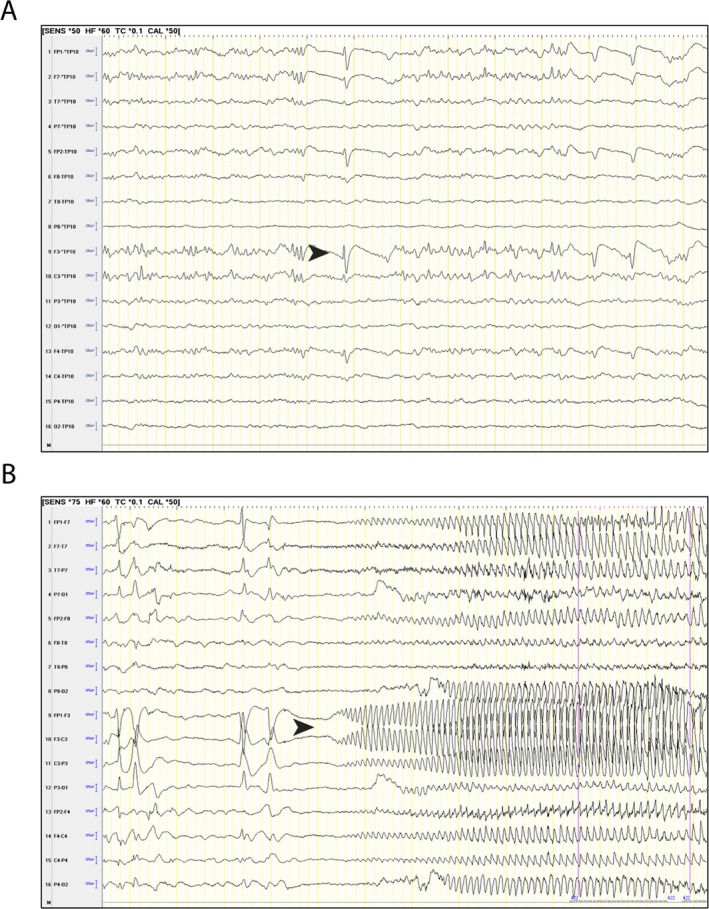
A, INTERICTAL: Left frontal spikes (arrow). B, ICTAL EEG: Left frontal (arrow). ICTAL SEMIOLOGY: right head version with bilateral asymmetric tonic posture

All patients were born at term; age at surgery ranged from 1 to 7 years. They all had frequent seizures refractory to pharmacological therapy and were thus referred for surgical treatment. Preoperative seizure frequency ranged from 1 to 5 a day, including both partial and generalized seizures. Engel outcome measures for these five patients showed clear reductions in seizure frequency at 1 year postsurgery. All cases had clear electrophysiological and semiological features that enabled specific surgical strategies to be reached on an individual basis, similar to the case of patient HME 4143, illustratively described herein. This patient's EEG showed asymmetric disorganization and accentuated basal activity in the left hemisphere along with epileptiform paroxysms of varying morphology and continuous incidence in the frontal and temporal regions of the left cerebral hemisphere (Table 1, Figure 1).

**Table 1:** Clinical and genetic findings in HME patients

Clinical characteristics	Patients				
	HME‐4143	HME‐4146	HME‐ 4149	HME‐6584	HME‐6593
Gender	M	F	M	M	M
Year of birth	2007	2012	2002	2002	2012
Epilepsy onset	3 m	9 m	3 m	8 m	3 m
Frequency of seizures (per month)/Pre‐Op	3	80	120	150	3
Neuropsychomotor development delay	Yes	Yes	Yes	Yes	Yes
Hemiparesis	Not	Not	Yes	Yes	Yes
Type of seizures	Focal to bilateral tonic‐ clonic	Focal	Generalized	Focal to bilateral tonic‐ clonic	Focal
Age at surgery (years)	5	2	6	13	4
EGG/ Pre‐op	Asymmetric disorganization of the accentuated base activity in the left hemisphere; epileptiform paroxysms of varying morphology of continuous incidence in the frontal and temporal cerebral regions of the left hemisphere.	Alternation theta in the region tempororolandica left; multifocal focal epileptiform paroxysms, polypone, acute wave and slow wave in frontotemporal‐ rolandic regions of the left cerebral hemisphere.	Asymmetry of the base activity, accentuated to the right; multifocal epileptiform paroxysms of acute tip‐wave type affecting poserior quadrant and right frontal region.	Increase in the frequency of discharges in the left cerebral hemisphere, with a theta and wider activity in the left parietal region and repetitive spikes in the frontal and left fronto‐polar regions.	Disorganized and asymmetric base activity; continuous epileptiform paroxysms in the left cerebral hemisphere, associated with slow activity.
Type of surgery	Left hemispherotomy	Left hemispherotomy	Right hemispherotomy	Left hemispherotomy	Left frontal lobectomy
Diagnosis and Histopathology	PMG, GA, NH, BC I	CG, CN, BC, GA, NH I	CD, BC, CN, DN I	DN, BC, CD, PMG I	DN, CD, BC, CG I
Engel 6 months	I	I	I	I	I
Engel 1 year	I	I	I	I	I
Pre‐op MRI	Axial and coronal MRI of FLAIR showing left and right cerebral cerebellar volume increase, with lateral ventricular dilatation and periventricular hypersignal.	Axial T2‐weighted and coronal sections in FLAIR of MRI showing evidence of signal change and morphology of spins throughout the left hemisphere.	Axial and coronal section of MRI in FLAIR showing right brain hemisphere enlargement, cortical thickening, lateral ventricular dilatation, and periventricular hypersignal	Axial and coronal MRI of FLAIR showing increased right brain hemisphere volume, lateral ventricular dilatation.	Axial and coronal section of MRI in FLAIR showing increased left cerebral hemisphere volume, cortical thickening, lateral ventricular dilatation, and periventricular hypersignal area.
Genetic screening method	WES/amplicon	WES/amplicon	WES/amplicon	Amplicon	Amplicon
Gene	*PIK3CA, DEPDC5*	*MTOR*	*PIK3CA*	*MTOR*	*PIK3CA*
Types of variants found	Missense, stop codon	Missense	Missense	Missense	Missense
HGVS	p.Cys420Arg p.Arg286Ter	p.Leu7105Phe	p.Glu542Lys	p.E642K	p.His1047Arg
ddPCR percentage of mutated cells	22% 5%	6%	11%	9.7%	13.1%
In silico predictions	Benign (PolyPhen) Pathogenic (MutationTaster) Benign (Sift) Pathogenic (PolyPhen) Pathogenic (MutationTaster) Pathogenic (Sift)	Pathogenic (PolyPhen) Pathogenic (MutationTaster) Pathogenic (Sift)	Pathogenic (PolyPhen) Pathogenic (MutationTaster) Benign (Sift)	Pathogenic (PolyPhen) Pathogenic (MutationTaster) Pathogenic (Sift)	Pathogenic (PolyPhen) Pathogenic (MutationTaster) Benign (Sift)

Abbreviations: BC, balloon cells; CD, cortical demyelination; CN, cytomegalic neurons; DN, dysmorphic neurons; GA, astrogliosis; NH, heterotopia; PMG, polymicrogyria.

**Figure 2:**
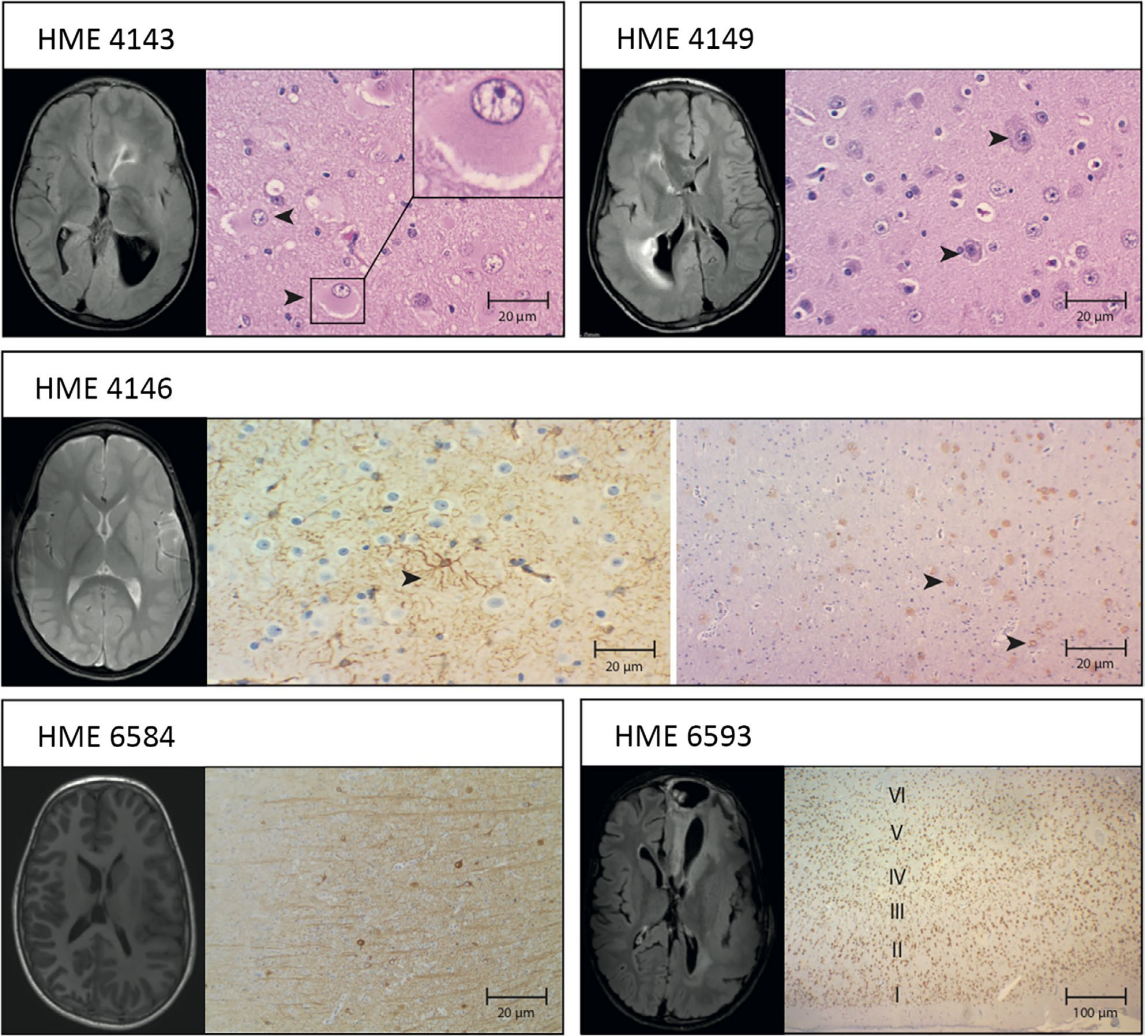
Somatic‐positive HME cases exhibit MRI and histopathological findings. A, Tissue shows the presence of balloon cells by coloration hematoxylin and eosin and pre‐op FLAIR MRI left and right cerebral cerebellar volume increase, with lateral ventricular dilatation and periventricular hypersignal areas. B, Tissue shows cytomegalic and dysmorphic neurons, with accumulation of Nissel's substance at the periphery coloration hematoxylin/eosin, and pre‐op FLAIR MRI shows right hemisphere enlargement, cortical thickening, lateral ventricular dilatation, and periventricular hypersignal areas. C, Astrogliosis immunostained to GFAP and pre‐op T2 MRI shows evidence of signal change and morphology throughout the left hemisphere. D, Tissue shows the presence of heterotopic neurons in the white matter neocortex transition, immunostained to NeuN, and (E) tissues show neurofilaments accumulating in the body of dysplastic neurons immunostained for NeuN, and pre‐op FLAIR MRI shows increased right brain hemisphere volume and lateral ventricular dilatation. F, Tissue shows disorganization of the cortical layers IV, V, VI with neuronal loss by immunostaining for NeuN, and pre‐op FLAIR MRI shows increased left cerebral hemisphere volume, cortical thickening, lateral ventricular dilatation, and periventricular hypersignal areas

As per the MRI scans, the right hemisphere was affected in two patients and the left hemisphere in three patients. Scans also showed that all patients had cortical malformations such as broad or flat gyri and shallow sulci (pachygyria or polymicrogyria) and white matter abnormalities (increased volume of white matter, advanced myelination) in the affected hemisphere (Table 1 and Figure 2). Most affected individuals show isolated forms of the disease, but HME is occasionally associated with neurocutaneous syndromes, including tuberous sclerosis, hypomelanosis of Ito, and Proteus syndrome. Nevertheless, none of our patients had neurocutaneous syndromes or somatic overgrowth other than the HME.

Histological and immunohistochemistry analyses showed disorganized cortical lamination, presence of balloon cells, Chaslin's gliosis, cytomegalic neurons, dysmorphic neurons, ectopic neurons, polymicrogyria, and intensely marked astrocytes with hypertrophic processes (Table 1 and Figure 2).

**Figure 3:**
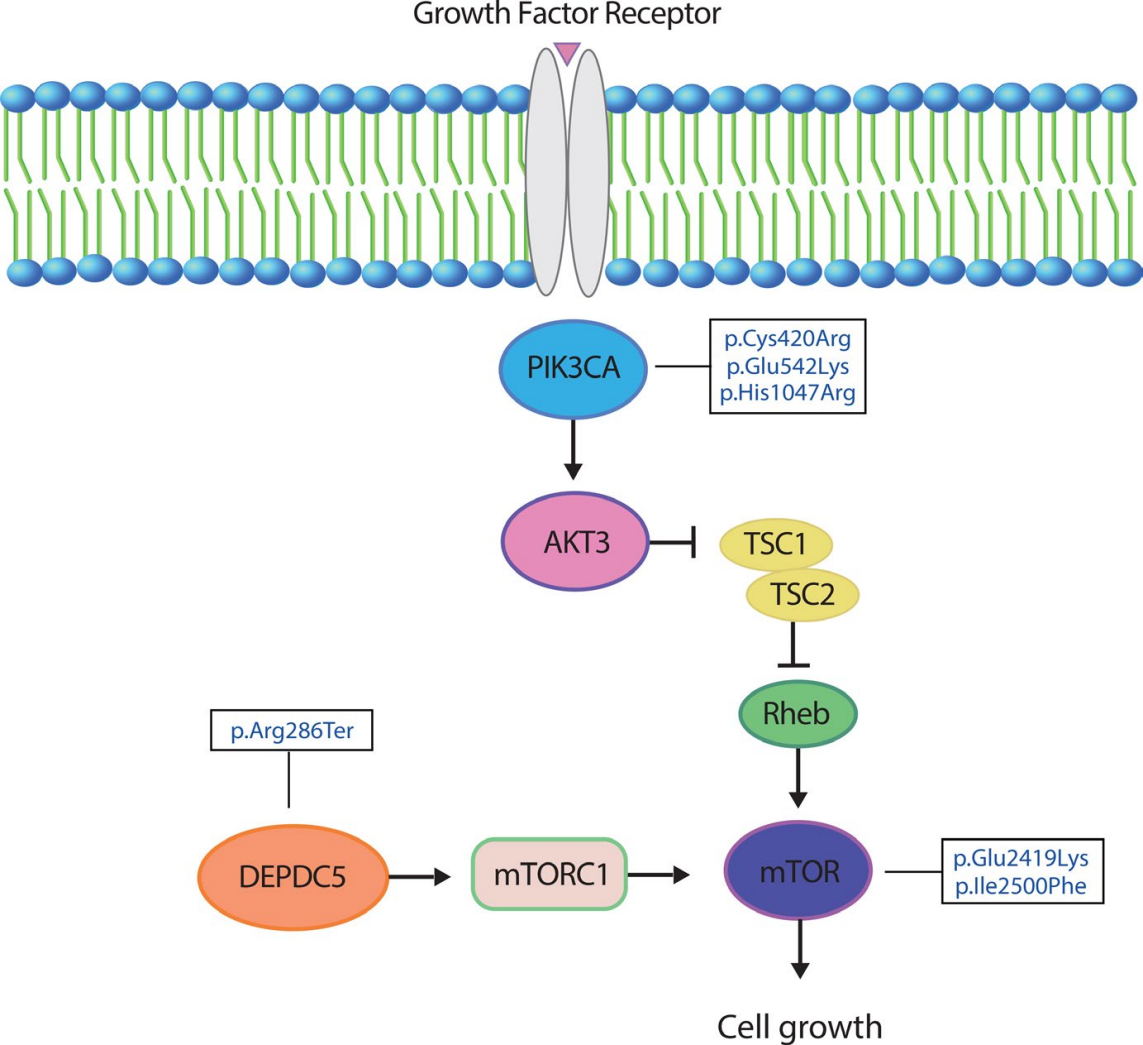
Schematic representation of the mTOR pathway annotated with pathogenic mutations identified in this study

To evaluate the role of somatic mTOR pathway variants in these patients, we performed WES and amplicon sequencing on paired DNA samples from brain and blood. In the three cases selected for WES analysis, a total of 840 variants were found. Protein variants were considered pathogenic if they were frameshift, in‐frame deletions, missense, stop gain, splice acceptor, and splice donor variants previously shown to be pathogenic by functional studies, or previously identified in HME or related syndromes. We then focused on the validation of variants found in three mTOR pathway genes that have been previously linked to malformations of cortical development, and on variant types leading to mTOR hyperactivation (gain of function in *MTOR* and *PIK3CA,* or loss of function in *DEPDC5).* The two samples that did not undergo WES were also screened for these variants. We validated four rare and protein‐truncating variants in *DEPDC5, MTOR,* and *PIK3CA* genes (Table 1). In HME 4143, we identified two variants: p.Cys420Arg (c.1258T > C) in *PIK3CA*, with a variant allele fraction of 22% by ddPCR validation, and p.Arg286Ter (c.856C > T) in *DEPDC5* with a 5% variant allele fraction by ddPCR. In HME 4146, we identified p.lle2500Phe (c.7498A > T) in *MTOR* with a variant allele fraction of 6% by ddPCR. For HME 4149, we found p.Glu542Lys (c.1624G > A) in *PIK3CA* with an 11% variant allele fraction by ddPCR. In the amplicon analysis for two other cases, we identified the missense variant p.Glu2419Lys (c.7255G > A) in *MTOR*, with an allele fraction of 9.7% by ddPCR and for HME 6584, and the missense variant p.His1047Arg (c.3140A > G) in *PIK3CA*, with a variant allele fraction of 13.1% by ddPCR (Table 1, Figure 3).

The missense variants p.Cys420Arg, p.Glu542Lys, and p.His1047Arg in *PIK3CA,* and lle2500Phe and p.Glu2419Lys in *MTOR* genes*,* are variants classified as presenting a moderate impact according to SnpEff annotation. All the variants in *MTOR* genes were also predicted as pathogenic and for the missense variants in *PIK3CA* gene that has been described as benign by the in silico tools. The variant p.Arg286Ter in *DEPDC5* gene was the only one classified as presenting high impact according to SnpEff, since it is a nonsense variant. p.Arg286Ter is also predicted as pathogenic by the in silico tools and is characterized as pathogenic by the ClinVar database (https://www.ncbi.nlm.nih.gov/clinvar/) (Table 1).

## 4 DISCUSSION

In the present study, we aim to add to the current understanding of which mTOR pathway variants may contribute to HME pathogenesis. Our results suggest that the presence of brain somatic variants, which have been increasingly reported in neurodevelopmental diseases associated with epilepsy, is associated with HME and the presence of seizures. Current understanding of developmental and genetic neuropathology implicates that FCD type IIb and HME can be included within the spectrum of a single disease. Constitutive activation of mTOR signaling represents a shared pathogenic mechanism in a group of developmental malformations that have histopathological and clinical features in common, such as epilepsy, autism, and other comorbidities. Also, from a neuropathological perspective, the most important distinction between hemimegalencephaly and FCD is their relative extent or the size/volume of the lesion. This difference is related to the onset of the postzygotic somatic mutation with activation of the mTOR pathway.^13^, ^14^ Thus, the characterization of variants in mTOR pathway genes raises the possibility that mTOR inhibitors could have wider therapeutic benefit for patients with focal epilepsy.^7^

Of the five studied cases, two presented with *MTOR* variants and three with missense *PIK3CA* variants*.* Those variants have been previously predicted as tentative pathogenic variants by in silico tools, except a mutation in *PIK3CA* which was described as benign. Even though these somatic variants have a low prevalence, we believe that they may be contributing to the genetic cause of HME in these patients. Dysregulation of the mTOR pathway has been implicated in many pathological conditions, including dysplasias of the central nervous system. Lee and colleagues^5^ performed WES on brain tissues and found variants in 6/20 HME patients, in genes of the mTOR pathway such as *AKT3*, *MTOR*, and *PIK3CA*; although they did not find the same variants in paired blood samples, they also suggested that these variants may affect this pathway and might be implicated in or even causing the disease.^5^, ^6^

Recent studies have reported on the prevalence of somatic variants of mTOR pathway genes in patients diagnosed with HME, showing that *AKT3* and *PIK3CA* might be mutated in 8%–10% of the studied cases.^6^, ^7^, ^15^ D’Gama et al, using deep sequencing of these mTOR genes, identified an etiology in 27/66 cases (41%).^16^ Rivière et al highlight the central role of PI3K/AKT de novo mutations in HME patients, and the power of massive parallel sequencing in the challenging context of phenotypic and genetic heterogeneity combined with postzygotic mosaicism mutations.^17^ In general, pathogenic variants in *PIK3CA* account for 6 to 25% of malformations of cortical development and approximately 31% of HME cases.^4^, ^6^, ^7^

Another study analyzed 446 tissue samples from 232 patients with intractable epilepsy with various brain pathologies, using deep sequencing of known epilepsy‐related genes (up to 28 genes), followed by confirmatory site‐specific amplicon sequencing.^18^ Pathogenic mutations of mTOR‐related genes were discovered in 31.9% (74/232). Baldassari et al enrolled 80 cases in a single‐center study and found pathogenic mutations in several mTOR pathway genes, providing a framework for efficient FCD and HME genetic testing, linking neuropathology to recent genetic discoveries, and emphasizing the usefulness of molecular assessment in the children with refractory epilepsy who are thus surgical candidates.^19^

More recently, *DEPDC5* variants have been detected in patients with focal epilepsy associated with malformations of cortical development. Of the five screened patients, one (HME 4143) had a nonsense variant, which was predicted as pathogenic by in silico tools and which is already characterized as pathogenic in ClinVar. This variant has been previously described as a candidate pathogenic variant associated with epilepsy, and further work is required to elucidate whether patients with epilepsy resulting from *DEPDC5* may be at increased risk of epilepsy‐related sudden death (SUDEP).^20^, ^21^

According to recent studies, *DEPDC5* variants are associated with a spectrum of focal epilepsy phenotypes, ranging from nonlesional familial focal epilepsies to malformation‐associated focal epileptic syndromes.^15^, ^20^ Also, variants in this gene may be associated with a higher rate of refractory epilepsies, as suggested by Baulac and colleagues,^20^ who reported that the majority of patients with refractory epilepsy presented with *DEPDC5* variants.

DEPDC5 is part of the GATOR1 complex, which inhibits the activity of mTORC1 under amino acid deprivation conditions. It is predicted that loss of function in GATOR1 genes will result in excessive activity of the mTORC1 kinase. Several studies assessed phosphorylation levels of the mTORC1 S6 substrate in brain samples of individuals with GATOR1‐related variants. Increased levels of S6 phosphorylation were observed in subjects with DCF IA, DCF IIA, and HME (even with histopathology of DCF IIA), confirming that pathogenic variants in GATOR1 genes are the cause of mTORC1 hyperactivity observed in these cases of CDF and HME.^22^, ^23^, ^24^, ^25^

The study by Marsan and colleagues showed that mTORC1 is hyperactivated in *DEPDC5* knockout mice and that a single prenatal injection of rapamycin could avoid its overall growth delay, demonstrating that embryonic lethality is caused by hyperactivation of mTORC1. Hughes et al (2017) corroborated these findings and reported blood vascular defects, as well as cortical and lymphatic developmental malformations underlying embryonic lethality in *DEPDC5* knockout rats.^26^, ^27^

Our study confirms the presence of *DEPDC5* variants in a patient diagnosed with HME and refractory epilepsy, but we believe that the refractory epilepsy should be treated with surgery even in the presence of a clear genetic etiology. The finding that *DEPDC5* is associated with cases of HME following hereditary focal epilepsy clarifies the evidence that *DEPDC5* acts as an inhibitor of mTORC1, and variants in *DEPDC5.*

may be responsible for structural changes in the cortex with the presence of dysmorphic cells due to hyperactivation of mTORC1.^20^, ^24^, ^26^, ^28^

Finally, we suggest that the somatic variants identified in HME may lead to malformations of cortical development by altering the mTOR signaling cascade. Consistent with this, positively regulated mTOR signaling was found in brain tissue of two of our patients (HME 6584 and HME 4146), in whom we identified somatic missense variants in *MTOR*. Other studies have also concluded that HME is caused by novel variants in the *PIK3CA*, *AKT3*, and *MTOR* genes, characterizing those variants as one of the molecular causes of HME.^5^

These findings reveal the existence of molecular biomarkers belonging to the mTOR pathway. These observations also suggest that HME represents a spectrum of neurodevelopmental disorders resulting from distinct progenitors that are determined at the time the variant occurs during brain development. One variant that occurs early in development may be expected to affect a large number of cells and result in a larger malformation, whereas the same variant occurring later in development could cause a minor malformation. However, identification of such somatic variants will require very high‐coverage next‐generation sequencing, ideally of affected brain tissue, given that the variant may be present in only a small fraction of cells.^7^, ^29^ Moving forward, it will be critical to perform such ultradeep sequencing, ideally using a targeted list of known and candidate genes, for HME and related disorders.

## CONFLICT OF INTEREST

We thank the support provided by the Research Support Foundation (FAEPA) of the University Hospital of Ribeirão Preto Medical School and the Center of Genomic Medicine, University Hospital, Ribeirão Preto Medical School (CMG/HCFMRP) for patient recruitment and sample preparation. This work was supported by grants from the Howard Hughes Medical Institute (to JGG), and Camila B. Garcia was recipient of a scholarship from the National Support Program for the Health Care of Persons with Disabilities (PRONAS/PCD) and Coordination of Improvement of Higher Education Personnel‐Brazil (CAPES). The authors confirm that they have read the journal's position on issues involved in ethical publication and affirm that this report is consistent with those guidelines.
